# Functional enrichment analysis reveals the involvement of DARS2 in multiple biological pathways and its potential as a therapeutic target in esophageal carcinoma

**DOI:** 10.18632/aging.205569

**Published:** 2024-02-20

**Authors:** Xu-Sheng Liu, Zi-Yue Liu, Dao-Bing Zeng, Jian Hu, Xuan-Long Chen, Jiao-Long Gu, Yan Gao, Zhi-Jun Pei

**Affiliations:** 1 Department of Nuclear Medicine, Hubei Provincial Clinical Research Center for Umbilical Cord Blood Hematopoietic Stem Cells, Taihe Hospital, Hubei University of Medicine, Shiyan 442000, Hubei, China; 2 Department of Critical Care Medicine, Danjiangkou First Hospital, Danjiangkou 420381, Hubei, China; 3 Department of Medical Ultrasound, Taihe Hospital, Hubei University of Medicine, Shiyan 442000, Hubei, China; 4 Department of Obstetrics and Gynecology, Taihe Hospital, Hubei University of Medicine, Shiyan 442000, Hubei, China

**Keywords:** DARS2, esophageal carcinoma, glycolysis, m6A, ceRNA

## Abstract

Objective: The enzyme Aspartyl tRNA synthetase 2 (DARS2) is a crucial enzyme in the mitochondrial tRNA synthesis pathway, playing a critical role in maintaining normal mitochondrial function and protein synthesis. However, the role of DARS2 in ESCA is unclear.

Materials and methods: Transcriptional data of pan-cancer and ESCA were downloaded from UCSC XENA, TCGA, and GEO databases to analyze the differential expression of DARS2 between tumor samples and normal samples, and its correlation with clinicopathological features of ESCA patients. R was used for GO, KEGG, and GSEA functional enrichment analysis of DARS2 co-expression and to analyze the connection of DARS2 with glycolysis and m6A-related genes. *In vitro* experiments were performed to assess the effects of interfering with DARS2 expression on ESCA cells. TarBase v.8, mirDIP, miRTarBase, ENCORI, and miRNet databases were used to analyze and construct a ceRNA network containing DARS2.

Results: DARS2 was overexpressed in various types of tumors. *In vitro* experiments confirmed that interfering with DARS2 expression significantly affected the proliferation, migration, apoptosis, cell cycle, and glycolysis of ESCA cells. DARS2 may be involved in multiple biological pathways related to tumor development. Furthermore, correlation and differential analysis revealed that DARS2 may regulate ESCA m6A modification through its interaction with METTL3 and YTHDF1. A ceRNA network containing DARS2, DLEU2/has-miR-30a-5p/DARS2, was successfully predicted and constructed.

Conclusions: Our findings reveal the upregulation of DARS2 in ESCA and its association with clinical features, glycolysis pathway, m6A modification, and ceRNA network. These discoveries provide valuable insights into the molecular mechanisms underlying ESCA.

## INTRODUCTION

Esophageal carcinoma (ESCA), one of the most prevalent gastrointestinal malignancies, is ranked seventh globally among the most common types of cancer and sixth among the leading causes of cancer-related mortality [1]. Furthermore, in the Chinese region, the incidence rate of esophageal cancer ranks fifth among new cases, exhibiting a steady upward trend over the years [2, 3]. Esophageal cancer is typically classified into two major types, namely, esophageal adenocarcinoma (ECA) and esophageal squamous cell carcinoma (ESCC), with the latter being the most predominant [4]. Due to the lack of effective techniques and strategies for early diagnosis and targeted treatment of ESCA, the five-year survival rate of ESCA patients is comparatively lower [5]. Consequently, regardless of histological subtypes, the absence of reliable biomarkers that can detect disease efficacy poses a significant drawback, necessitating the identification of such biomarkers in order to alleviate human suffering.

The enzyme Aspartyl tRNA synthetase 2 (DARS2) is a mitochondrial enzyme [6], and previous studies have reported its crucial role in the development of bladder cancer [7], lung adenocarcinoma [8, 9], ovarian cancer [10], and hepatocarcinogenesis [11]. While the impact of DARS2 has been explored in other types of cancer, its exact function in ESCA remains unclear. Cancer is characterized by uncontrolled proliferation, invasion and metastasis of tumor cells. The dysregulation of signaling pathways involved in cell proliferation, migration, and metabolism plays a crucial role in tumor development and progression [5, 12–14]. The growing evidence suggests that the abnormal metabolism of cancer cells, especially the reliance on glycolysis even in the presence of oxygen, the Warburg effect, plays a crucial role in tumor development and therapy resistance [15–19]. Moreover, recent studies have revealed the critical role of RNA modifications, such as N6-methyladenosine (m6A), in regulating cancer cell behavior, including proliferation, stemness, and drug resistance [20]. The ceRNA network (competitive endogenous RNA network) refers to a regulatory network formed by the interaction of multiple non-coding RNAs (ncRNAs). In this network, multiple ncRNAs, including long non-coding RNAs (lncRNA), circular RNAs (circRNA), and pseudogenes, compete to bind to common microRNAs (miRNAs), thereby influencing the regulatory effect of these miRNAs on other target RNAs. Existing studies have demonstrated that the ceRNA network plays a crucial role in the occurrence and progression of tumors [21–23]. Therefore, further elucidating the interaction between DARS2 and metabolic, epigenetic, and ceRNA network in ESCA can help develop new diagnostic and therapeutic methods.

In this study, we used a public database and *in vitro* experiments to explore DARS2 expression with ESCA, in order to investigate the impact of DARS2 expression on the proliferation, cell cycle, apoptosis, migration, and glycolysis of ESCA cells. Furthermore, an analysis on the potential correlation between DARS2 expression, and m6A as well as ceRNA network in ESCA was conducted, which provides novel targets and personalized treatment strategies for the management of this disease.

## MATERIALS AND METHODS

### Bioinformatics analysis of DARS2 expression

The present study utilized the TCGA-GTEx pan-cancer datasets obtained from UCSC XENA (https://xenabrowser.net/datapages/) that comprised of RNAseq data in transcripts per million (TPM) format from The Cancer Genome Atlas (TCGA) and the Genotype-Tissue Expression (GTEx) [24] that have undergone uniform processing via the Toil process [25]. We comprehensively analyzed the expression levels of DARS2 across 33 distinct tumor types. Meanwhile, we retrieved and curated the RNAseq data in TPM format of the TCGA ESCA dataset from the TCGA database (https://portal.gdc.cancer.gov) [26] to investigate the differential expression of DARS2 between tumor and normal groups, as well as between cancer samples and matched normal samples. Moreover, to further confirm the expression differences of DARS2 between ESCA and normal samples, we downloaded and analyzed the GSE20347, GSE38129, and GSE45670 datasets from the Gene Expression Omnibus (GEO, https://www.ncbi.nlm.nih.gov/geo/) database [27] using the GEOquery package [28]. In addition, we evaluated the diagnostic value of DARS2 expression in ESCA using the ROC curve analysis. Finally, we further investigated the relationship between the expression levels of DARS2 and the clinical pathological characteristics of ESCA patients. Finally, we conducted an analysis of the TCGA ESCA and GSE45670 datasets to investigate the potential relationship of DARS2 expression with glycolysis and m6A-related genes. The correlation analysis module of GEPIA online database (http://gepia2.cancer-pku.cn/#correlation) was used to explore the relationship between DARS2 expression and glycolysis and m6A signatures in ESCA. The glycolysis [29] and m6A [20] related gene lists used in this study were referenced from previous research.

### Functional enrichment analysis of DARS2 in ESCA

The R software package was utilized to analyze the TCGA ESCA dataset and investigate the co-expression of genes that were associated with DARS2 expression. The statistical correlation was verified using Pearson correlation coefficient, and the ggplot2 package of R software was employed to generate the volcano map and heat map for visualization. The co-expressed genes were analyzed using the clusterProfiler package (version 3.14.3) [30] for gene ontology (GO) function and the Kyoto Encyclopedia of Genes and Genomes (KEGG) pathway, and the data were visualized using the ggplot2 package.

We extracted the data of DARS2 from the TCGA ESCA dataset and divided it into high and low expression groups based on the expression level of DARS2. Then, the original counts matrix of the TCGA ESCA dataset was subjected to differential analysis using the DESeq2 package [31], aiming to obtain all differentially expressed genes (DEGs). ClusterProfiler software package (version 3.14.3) was utilized to conduct Gene Set Enrichment Analysis (GSEA, www.gsea-msigdb.org/gsea/index.jsp) [32] analysis on all DEGs in order to scrutinize potential enrichment of these genes in biologically meaningful processes. The reference gene set is c2.cp.all.v2022.1.Hs.symbols.gmt.

### Cell culture and treatment

The human ESCA cell line Kyse150 cells were purchased from the BeNa Culture Preservation Center (BNCC359343, BNCC). Human esophageal epithelial cells (HET-1A cells) were obtained from the Cell Bank of Chinese Academy of Sciences (Shanghai, China). The culture medium used for cell culture includes RPMI-1640 complete medium (KGM31800S, KeyGEN) and RPMI-1640 incomplete medium (KGM31800N, KeyGEN). Transfection of cells with DARS2 siRNA was carried out as per the manufacturer’s instructions using Lipofectamine 3000 transfection reagent (L300015, Invitrogen). The detailed siRNA sequence is shown in Table 1.

**Table 1:** The sequences of siRNAs used in this study.

**Gene**	**Sense**	**Antisense**
si-DARS2#1	AGGUGAGAUUGAAAUCAAATT	UUUGAUUUCAAUCUCACCUTT
si-DARS2#2	GGAAUGUGCUGACCUUCUATT	UAGAAGGUCAGCACAUUCCTT
NC	UUCUCCGAACGUGUCACGUTT	ACGUGACACGUUCGGAGAATT

### Extraction of RNA and qRT-PCR analysis

Extracting total RNA from ESCA cell lines using TRIzon Reagent (CW0580S, CWBIO). For cDNA synthesis, HiScript II Q RT SuperMix for qPCR (R223-01, Vazyme) was employed. Real-time quantitative PCR (qRT-PCR) analysis was conducted using the CFX Connect™ system (Bio-Rad). The relative expression levels were determined using the 2−ΔΔCt method. We used β-Actin as a standardized internal control. The detailed primer sequence is shown in Supplementary Table 1.

### EdU proliferation assay

The proliferative activity of ESCA cells transfected with siRNA was detected using the EdU kit (C0078S, Beyotime). For detailed protocols, see our previous studies [33].

### CCK-8 assay for cellular viability

According to the manufacturer’s instructions, we incubated the cultured cells with the CCK-8 reagent (KGA317, KeyGen) for evaluating cell metabolic activity through the activity of cellular reductase. Lastly, we measured the absorbance values using a spectrophotometer. The changes in absorbance values reflected the proliferation activity of cells, allowing for a quantitative assessment of cell proliferation extent.

### Clone formation assay

The transfected individual cells were evenly dispersed in a culture dish, and after a week of cultivation, the cell population developed into visible clones. Following the protocol, the cells were washed with PBS and fixed with polyformaldehyde for subsequent imaging. The cloning efficiency could be quantitatively assessed by counting the number of clones to evaluate cell proliferation rate.

### Wound healing assay

Inoculate the transfected cells into a six well culture plate until they achieve 90% fusion. Using 200 μL tip of the pipette scrapes the cells. Observe the migration of cells under a microscope at 0, 24, and 48 hours. Quantify scratch areas using ImageJ software.

### Apoptosis assay

The Annexin V-FITC/PI Apoptosis Detection Kit (AP101-100Kit, MULTI SCIENCES) was used to detect apoptosis. Apoptosis was detected by NovoCyte™ flow cytometry (NovoCell 2060R, ACEA Biosciences Inc.) at 488 nm.

### Cell cycle assay

The cultivated cells were stained with a cell cycle staining kit (CCS012, MULTISCIENCES) for analysis. Flow cytometry was employed for cellular analysis.

### 2-NBDG uptake assay

Cells were inoculated into 96-well plates at a density of 2 × 10^4 cells per well. Transfection with siRNA was performed and after 24 hours, the cells were washed with PBS. Subsequently, the cells were incubated with 50 μM of 2-NBDG (HY-116215, MCE) in glucose-free DMEM for 30 minutes at 37° C with 5% CO_2_. Following the incubation period, the cells were washed three times with warm PBS. The mean fluorescence intensity (MFI) of the 2-NBDG was quantified by NovoCyte™ Flow cytometry.

### Lactic acid production

The lactate levels in the culture medium were measured using the Lactate Colorimetric Assay Kit (E-BC-K044-M, Elabscience), as per the manufacturer’s instructions.

According to the manufacturer’s instructions, we measured the absorbance at 530 nm.

### CeRNA network analysis

We predicted potential miRNAs targeting DARS2 by utilizing the TarBase v.8 (https://dianalab.e-ce.uth.gr/html/diana/web/index.php?r=tarbasev8) [34], mirDIP (http://ophid.utoronto.ca/mirDIP/index.jsp#r) [35], and miRTarBase (https://mirtarbase.cuhk.edu.cn/~miRTarBase/miRTarBase_2022/php/index.php) [36] platforms, and confirmed the final target miRNAs through differential expression analysis. Similarly, we predicted lncRNAs targeting the target miRNAs using the ENCORI (https://rnasysu.com/encori/index.php) [37] and miRNet (https://www.mirnet.ca/miRNet/home.xhtml) [38] platforms, and identified the final target lncRNAs through differential expression analysis. Finally, based on the ceRNA hypothesis, we constructed a ceRNA network. We employed the RNAHybrid online tool to predict potential binding sites between mRNA-miRNA and lncRNA-miRNA interactions.

### Statistics analysis

We performed statistical analysis and plotted graphs using Xiantao online database tool (https://www.xiantaozi.com/) and GraphPad Prism statistical software. For group comparisons, T test or Wilcoxon rank sum test was employed. One-way ANOVA or Two-way ANOVA was used for multiple group comparisons. A P-value <0.05 was considered statistically significant. “ns” indicates no significance; *, p < 0.05; **, p < 0.01; ***, p < 0.001; ****, p < 0.0001.

### Availability of data and materials

The datasets generated during and/or analysed during the current study are available from the corresponding author upon reasonable request.

## RESULTS

### Bioinformatics analysis of DARS2 expression

**Figure 1:**
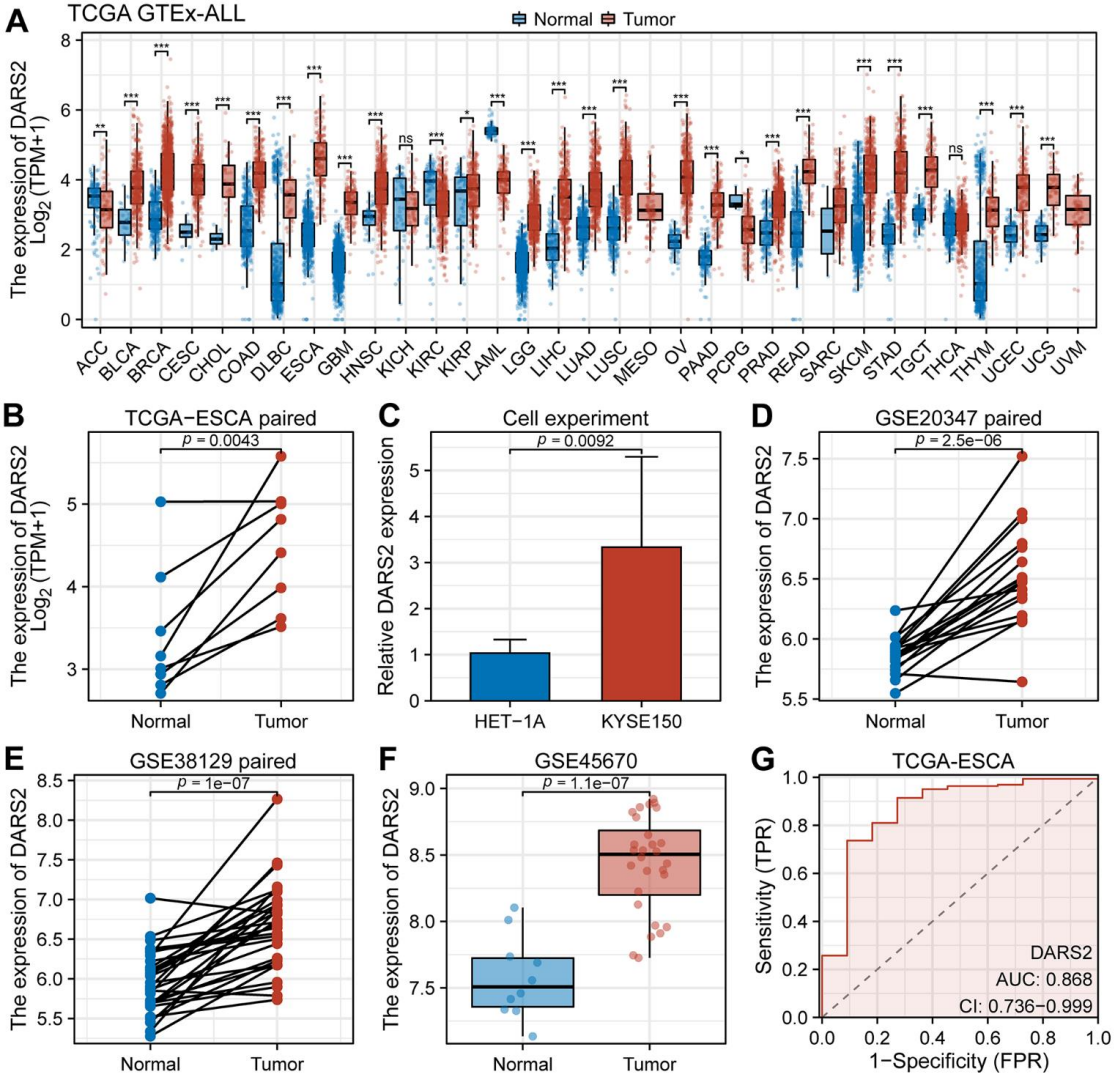
**Bioinformatics analysis of DARS2 expression.** (**A**) The expression level of DARS2 was assessed by analyzing the TCGA-GTEx pan-cancer datasets. (**B**) In the TCGA ESCA dataset, the differential expression of DARS2 between cancer samples and paired normal samples. (**C**) Cell experiments evaluated differences in DARS2 expression. (**D**–**F**) The variation in DARS2 expression was investigated between ESCA cancer tissue and normal tissue in the GSE20347, GSE38129, and GSE45670 datasets. (**G**) Receiver operating characteristic (ROC) curve analysis was conducted to evaluate the utility of DARS2 as a diagnostic marker for ESCA. *p < 0.05; **p < 0.01; ***p < 0.001; ns, no significance.

In order to determine the expression profile of DARS2 in the majority of common cancers, we performed a pan-cancer analysis based on the TCGA and GTEx databases. The results showed a significant increase in the expression level of DARS2 in Bladder Urothelial Carcinoma (BLCA), Breast invasive carcinoma (BRCA), Cervical squamous cell carcinoma and endocervical adenocarcinoma (CESC), Cholangiocarcinoma (CHOL), Colon adenocarcinoma (COAD), Lymphoid Neoplasm Diffuse Large B-cell Lymphoma (DLBC), Esophageal carcinoma (ESCA), Glioblastoma multiforme (GBM), Head and Neck squamous cell carcinoma (HNSC), Kidney renal papillary cell carcinoma (KIRP), Brain Lower Grade Glioma (LGG), Liver hepatocellular carcinoma (LIHC), Lung adenocarcinoma (LUAD), Lung squamous cell carcinoma (LUSC), Ovarian serous cystadenocarcinoma (OV), Pancreatic adenocarcinoma (PAAD), Prostate adenocarcinoma (PRAD), Rectum adenocarcinoma (READ), Skin Cutaneous Melanoma (SKCM), Stomach adenocarcinoma (STAD), Testicular Germ Cell Tumors (TGCT), Thymoma (THYM), Uterine Corpus Endometrial Carcinoma (UCEC) and Uterine Carcinosarcoma (UCS) compared to the normal group, while its expression level was significantly decreased in Adrenocortical carcinoma (ACC), Kidney renal clear cell carcinoma (KIRC), Acute Myeloid Leukemia (LAML) and Pheochromocytoma and Paraganglioma (PCPG) (Figure 1A, p < 0.05).

**Figure 2:**
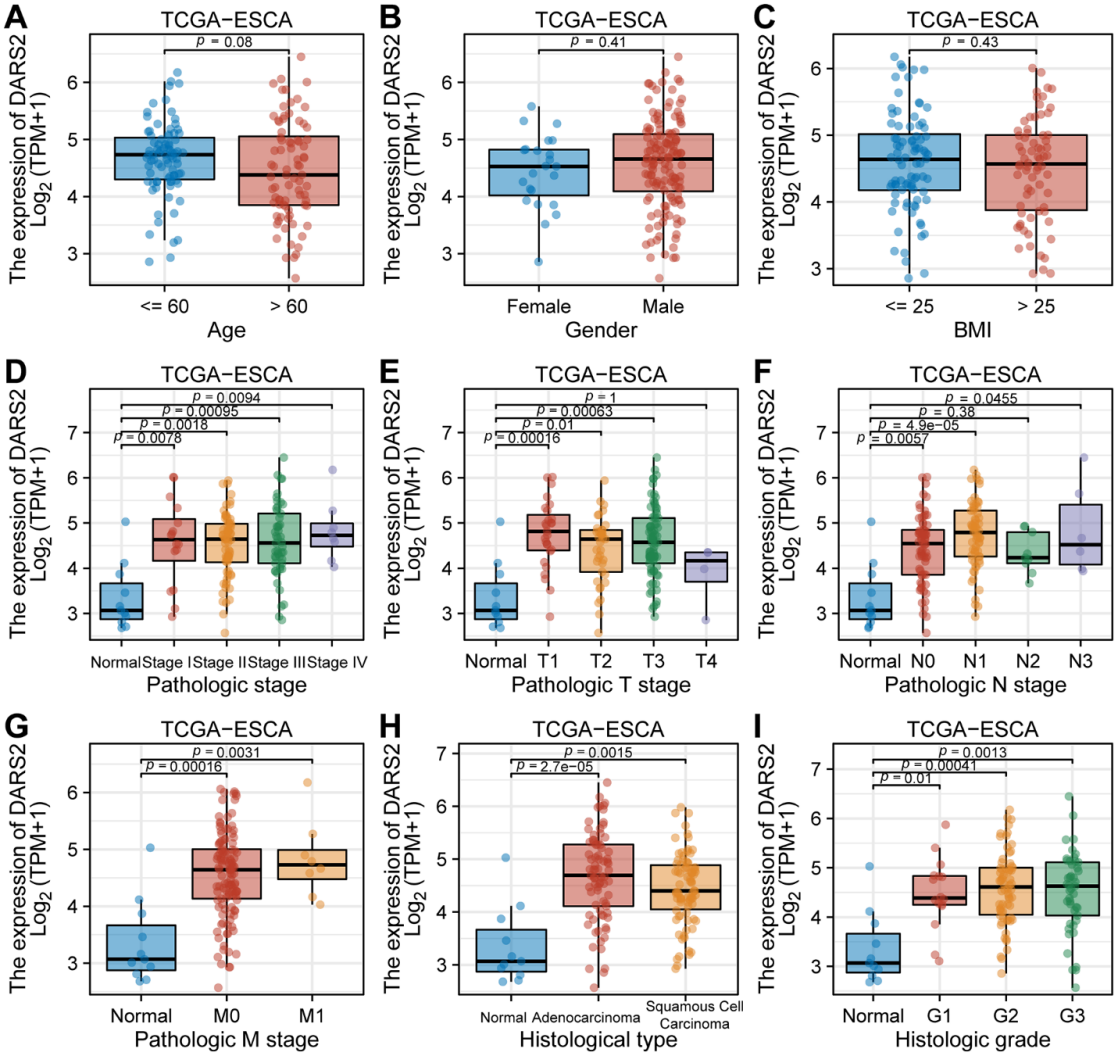
**The correlation between DARS2 expression and clinicopathological parameters in patients with esophageal carcinoma (ESCA).** The correlation between the expression level of DARS2 and (**A**) age, (**B**) gender, (**C**) BMI, (**D**) pathologic stage, (**E**) T stage, (**F**) N stage, (**G**) M stage, (**H**) histological staging and (**I**) histologic grade.

In the TCGA ESCA dataset, elevated expression of DARS2 was observed compared to the paired control normal samples (Figure 1B). The results of cell experiments revealed that the expression of DARS2 was significantly increased in human esophageal cancer cell lines compared to human normal esophageal epithelial cells (Figure 1C). Analysis of the GSE20347, GSE38129, and GSE45670 datasets demonstrated significantly higher expression levels of DARS2 in ESCA samples compared to the control group (Figure 1D–1F). In order to assess the diagnostic potential of DARS2 in ESCA, ROC curve analysis was conducted to further evaluate its performance. The results of the ROC analysis demonstrated that DARS2 exhibited significant potential in predicting ESCA, with notable accuracy, reflected by an AUC value of 0.868 (95% confidence interval [CI]: 0.736-0.999) (Figure 1G). To further elucidate the potential clinical significance of DARS2, a comprehensive analysis of clinical data from TCGA ESCA samples was performed (Figure 2). The research findings revealed that DARS2 expression was elevated in tumor samples of all stages (I, II, III, and IV) compared to the normal group (Figure 2D, p < 0.05). In terms of T staging, DARS2 expression was higher in tumor samples of T1, T2, and T3 stages compared to the normal group (Figure 2E, p < 0.05). Regarding N staging, DARS2 expression was higher in tumor samples of N0, N1, and N3 stages compared to the normal group (Figure 2F, p < 0.05). In terms of M staging, DARS2 expression was higher in tumor samples of M0 and M1 stages compared to the normal group (Figure 2G, p < 0.05). Additionally, DARS2 expression was higher in samples of ECA and ESCC compared to the normal group in the histological type of analysis (Figure 2H, p < 0.05). Furthermore, in the histological grade analysis, DARS2 expression was higher in tumor samples of G1, G2, and G3 stages compared to the normal group (Figure 2I, p < 0.05). However, there was no statistically significant difference in DARS2 expression with respect to age, gender, and BMI index in tumor patients (Figure 2A–2C, p > 0.05).

### Functional enrichment analysis of DARS2 in ESCA

**Figure 3:**
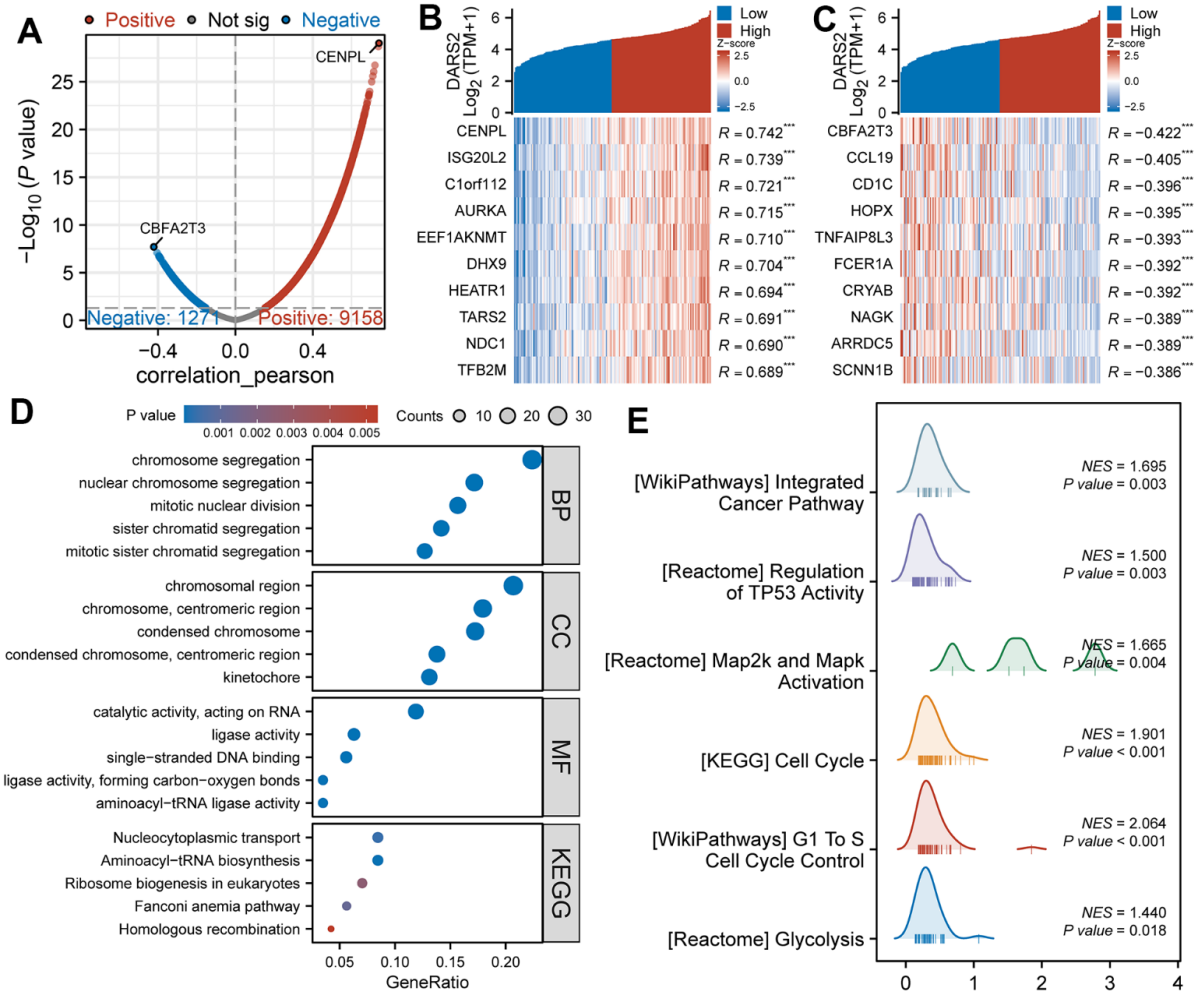
**Functional enrichment analysis of DARS2 in ESCA.** (**A**) The volcano plot exhibits the genes significantly correlated with DARS2 expression in the TCGA ESCA dataset. (**B**, **C**) Heatmap was used to visually display the top 10 most significant genes positively and negatively correlated with DARS2 expression. (**D**) Enrichment analysis was performed on co-expression genes of DARS2 using Gene Ontology (GO) terms and the Kyoto Encyclopedia of Genes and Genomes (KEGG) pathway. (**E**) GSEA analysis revealed DARS2 related pathways. *p < 0.05; **p < 0.01; ***p < 0.001; ns, no significance.

We performed Pearson correlation coefficient analysis to examine the correlation between DARS2 expression and other molecules in the TCGA ESCA dataset, specifically focusing on genes categorized as “coding proteins”. When selecting a threshold of p < 0.05, we identified a total of 9158 genes that showed a positive correlation with DARS2 expression, and 1271 genes that showed a negative correlation (Figure 3A). Among these genes, CENPL exhibited the highest positive correlation coefficient with DARS2, while CBFA2T3 exhibited the highest negative correlation coefficient. As illustrated in Figures 3B, 3C, the heatmaps were used to visually display the top 10 most significant genes positively and negatively correlated with DARS2 expression.

GO function and KEGG pathway enrichment analysis were conducted on co-expressed genes associated with DARS2 expression using the R software package. At a correlation coefficient above 0.6 and a significance level of p < 0.05, the co-expression of DARS2 resulted in involvement of a total of 332 biological processes (GO-BP), 69 cellular components (GO-CC), 72 molecular functions (GO-MF), and 5 KEGGs (p < 0.05). The bubble plot illustrates the top five most relevant groups of data in the order of GO-BP, GO-CC, GO-MF, and KEGG. GO functional annotation showed that the DARS2 co-expressed genes were mainly involved in chromosome segregation, chromosome regions, and catalytic activity, acting on RNA. KEGG pathway analysis reveals that the co-expression of DARS2 is predominantly associated with the Nucleocytoplasmic transport (Figure 3D).

To further investigate the potential functions of DARS2, we performed GSEA. Initially, the TCGA ESCA dataset was divided into high and low expression groups based on the expression levels of DARS2 to identify DEGs. The GSEA results revealed a total of 651 gene sets under the conditions of FDR (q-value) < 0.25 and p < 0.05. These gene sets mainly included WP_INTEGRATED_CANCER_PATHWAY (NES =1.70, p < 0.05), REACTOME_REGULATION_OF_TP53_ACTIVITY (NES = 1.50, p < 0.05), REACTOME_MAP2K_AND_MAPK_ACTIVATION (NES = 1.67, p < 0.05), KEGG_CELL_CYCLE (NES = 1.90, p < 0.05), WP_G1_TO_S_CELL_CYCLE_CONTROL (NES = 2.06, p < 0.05), and REACTOME_GLYCOLYSIS (NES = 1.44, p < 0.05) (Figure 3E).

### Decreased DARS2 affects cell proliferation and migration

**Figure 4:**
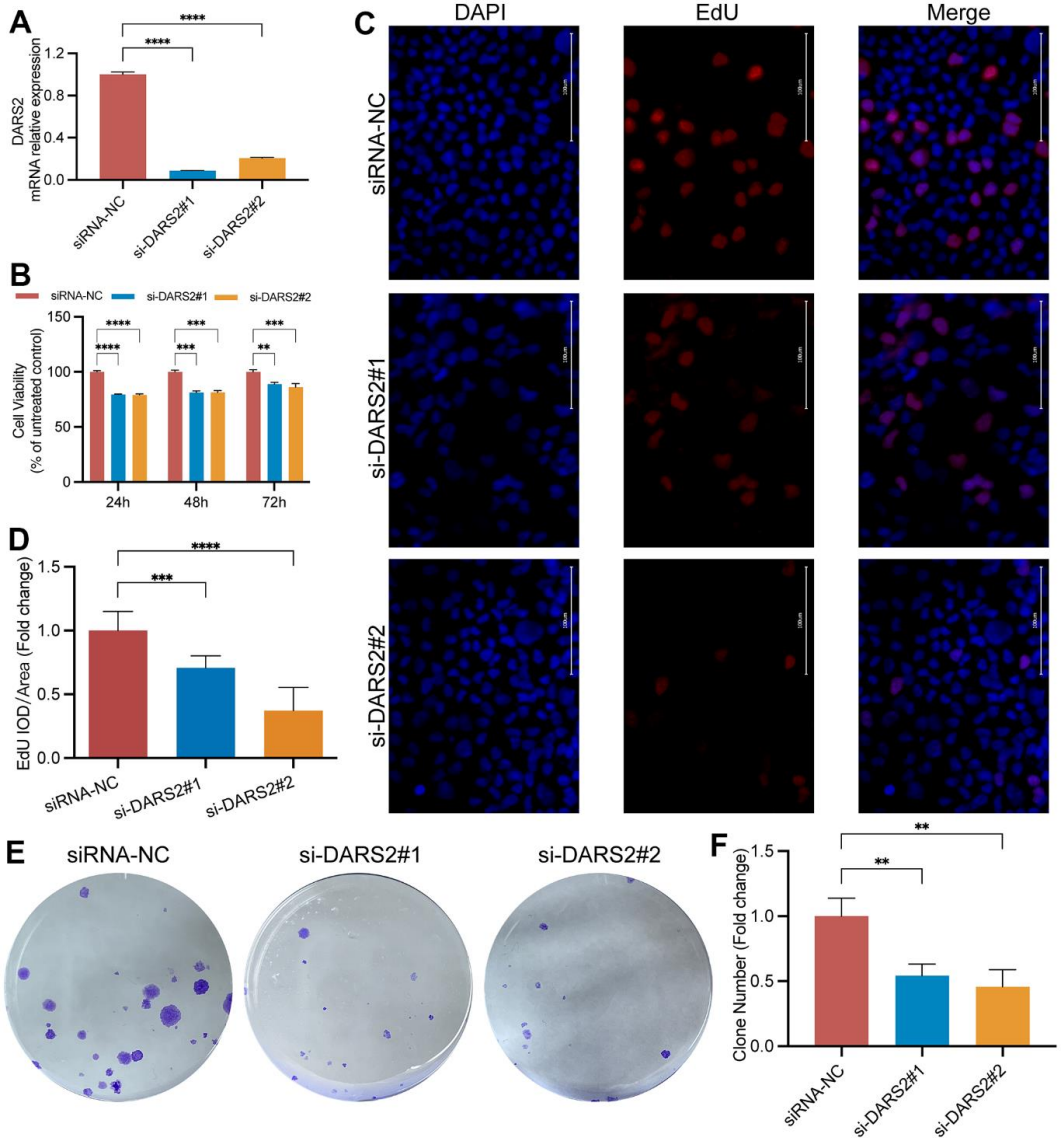
**DARS2 knockdown inhibits ESCA cell proliferation.** (**A**) The interference efficiency of two siRNAs was confirmed by qRT-PCR experiments. The results of CCK-8 assay (**B**), EdU proliferation experiments (**C**, **D**), and colony formation assay (**E**, **F**) demonstrated a significant decrease in cell proliferation activity in the experimental group compared to the control group. *p < 0.05; **p < 0.01; ***p < 0.001; ns, no significance.

**Figure 5:**
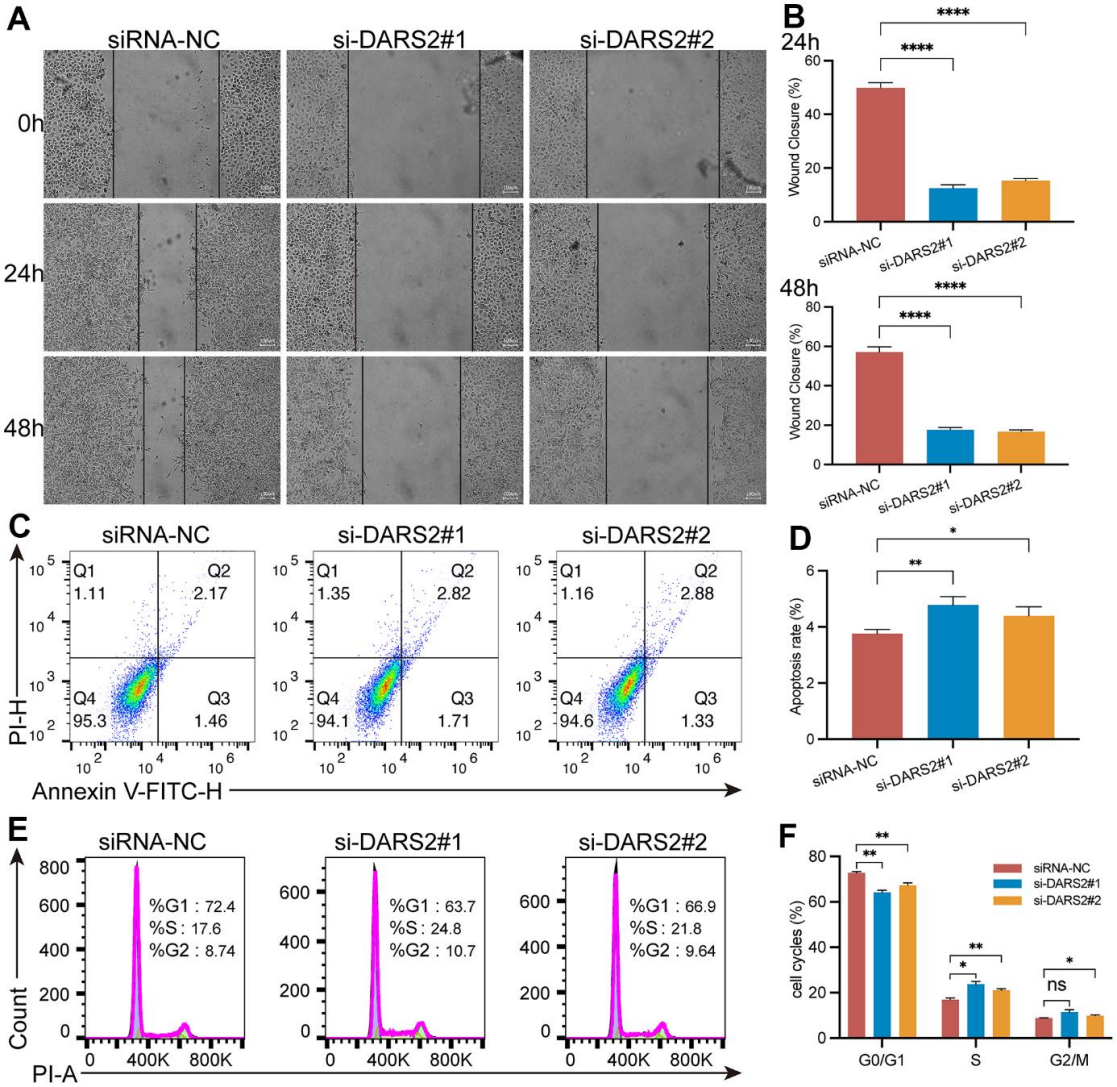
**DARS2 knockdown affects wound healing, apoptosis and cell cycle in ESCA cells.** (**A**, **B**) The analysis of wound healing in the present study revealed a significant reduction in wound healing rate upon downregulation of DARS2 expression. (**C**, **D**) Furthermore, the results demonstrated that downregulation of DARS2 expression significantly increased the number of apoptotic cells in the experimental group. (**E**, **F**) Flow cytometry analysis revealed a significant arrest of tumor cells in the S phase and G2/M phase in the experimental group compared to the control group. *p < 0.05; **p < 0.01; ***p < 0.001; ns, no significance.

To verify the potential inhibitory effect of DARS2 knockdown on ESCA cells, we conducted a series of experiments *in vitro*. Firstly, we conducted qRT-PCR experiments, which showed that the mRNA expression level of DARS2 was significantly reduced after knockdown (Figure 4A, p < 0.05). Subsequently, we conducted CCK-8 experiments and EDU proliferation experiments to determine cell activity and cell survival rate. In the CCK-8 assay, the cellular viability of the DARS2 knockdown groups was significantly reduced compared to that of the control group (Figure 4B, p < 0.05). In the EDU proliferation experiment, the cell survival rates of the two si-DARS2 groups significantly decreased compared to the control group (Figure 4C, 4D, p < 0.05). Finally, we conducted colony formation experiments to further validate our hypothesis, and the results showed that knocking down DARS2 resulted in a decrease in cell proliferation ability (Figure 4E, 4F, p < 0.05). It has been further confirmed that inhibition of DARS2 expression can significantly suppress the proliferation of ESCA tumor cells. Next, we used wound healing to further verify that DARS2 inhibition can reduce cell migration. In wound healing measurements, knocking down the expression of DARS2 can significantly inhibit wound healing rate (Figure 5A, 5B, p < 0.05). Meanwhile, the results from flow cytometry demonstrated a noteworthy elevation in the quantity of apoptotic cells in both si-DARS2 experimental groups (Figure 5C, 5D, p < 0.05). Flow cytometry analysis revealed that compared to the control group, tumor cells in the experimental group were found to be arrested in the S phase and G2/M phase (Figure 5E, 5F, p < 0.05), suggesting that inhibition of DARS2 expression may disrupt the normal cell cycle progression in tumor cells.

### Effects of DARS2 knockdown on ESCA glycolysis

**Figure 6:**
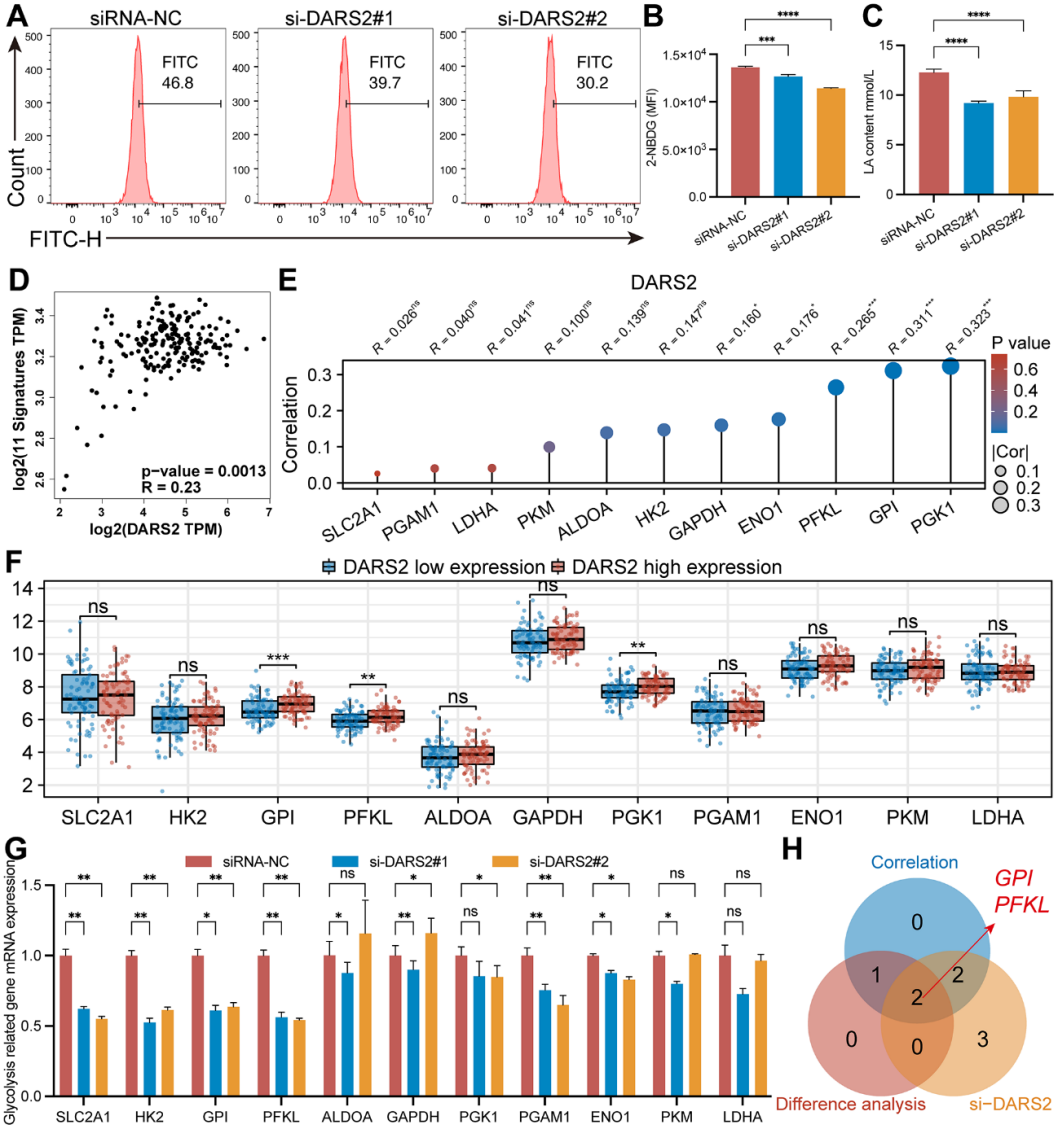
**Effects of DARS2 knockdown on ESCA glycolysis.** (**A**, **B**) Knocking down DARS2 significantly reduces the uptake of 2-NBDG in ESCA cells. (**C**) DARS2 knockdown significantly reduces lactate production in ESCA cells. (**D**) GEPIA online database analysis revealed that DARS2 expression was significantly positively correlated with glycolysis signatures in ESCA. (**E**) The correlation between 11 glycolysis-related genes and DARS2 expression in the TCGA ESCA dataset. (**F**) Analyzing the expression differences of 11 glycolysis-related genes between the DARS2 high and low expression groups in the TCGA ESCA dataset. (**G**) The expression differences of 11 glycolysis-related genes between the experimental group and the control group were analyzed using qRT-PCR experiments. (**H**) The Venn diagram illustrates the genes that satisfy the aforementioned three conditions, including GPI and PFKL. *p < 0.05; **p < 0.01; ***p < 0.001; ns, no significance.

Previous GSEA analysis revealed enrichment of glycolysis-related pathways. Subsequently, glucose uptake experiments were conducted, indicating a decrease in glucose uptake capacity in the si-DARS2 groups compared to the control group (Figure 6A, 6B, p < 0.05). Furthermore, lactate production experiments demonstrated significantly lower lactate production in ESCA tumor cells after DARS2 interference (Figure 6C, p < 0.05). GEPIA online database analysis revealed that DARS2 expression was significantly positively correlated with glycolysis signatures in ESCA (Figure 6D, p < 0.05). To further explore the potential association between DARS2 expression and glycolysis, we analyzed the correlation between 11 glycolysis-related genes and DARS2 expression in the TCGA ESCA dataset. The results showed a positive correlation between DARS2 expression and five glycolysis-related genes, namely PGK1, GPI, PFKL, ENO1, and GAPDH (Figure 6E, p < 0.05). Additionally, based on DARS2 expression, we divided the TCGA ESCA dataset into high and low expression groups, further analysis revealed significantly higher expression of three glycolysis-related genes (PGK1, PFKL, and GPI) in the high DARS2 expression group compared to the low DARS2 expression group (Figure 6F, p < 0.05). Finally, qRT-PCR experiments were performed to examine the expression of 11 glycolysis-related genes in ESCA cells transfected with si-DARS2, which showed that SLC2A1, HK2, GPI, PFKL, PGAM1, and ENO1 genes were significantly lower in both si-DARS2 groups compared to the control group (Figure 6G, p < 0.05). However, ALDOA, GAPDH, PKG1, and PKM genes only showed a downward trend in one si-RNA group. Although the LDHA gene exhibited a downward trend in both si-DARS2 groups, no statistical difference was observed. The Venn diagram illustrates the genes that satisfy the aforementioned three conditions, including GPI and PFKL (Figure 6H).

### Correlation between DARS2 expression and m6A modification in ESCA

**Figure 7:**
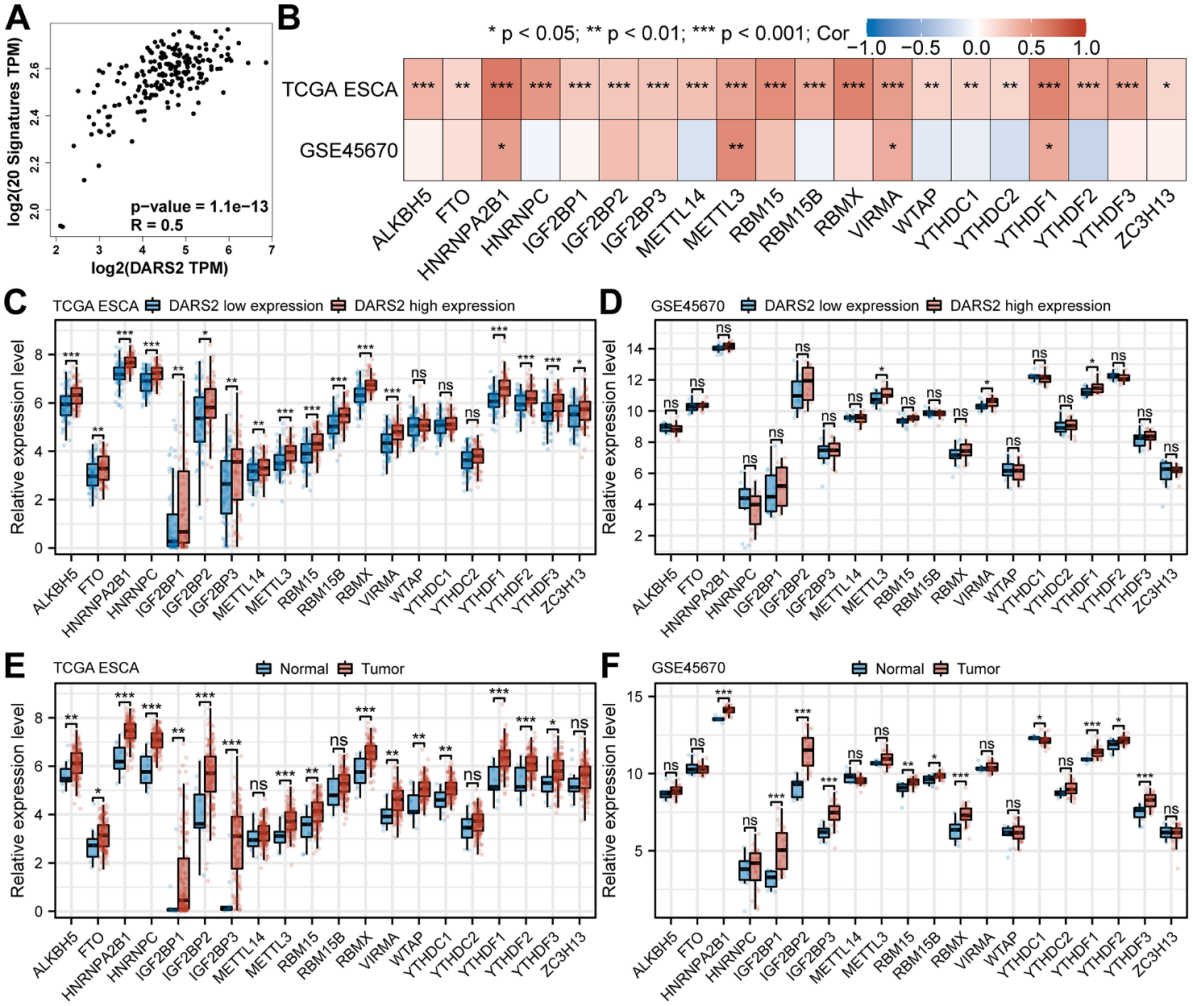
**Correlation between DARS2 expression and m6A modification in ESCA.** (**A**) GEPIA online database analysis revealed that DARS2 expression was significantly positively correlated with m6A signatures in ESCA. (**B**) The association between DARS2 expression and the expression of m6A-related genes in the TCGA ESCA and GSE45670 datasets. (**C**, **D**) The differential expression of 20 m6A-related genes between the high and low DARS2 expression groups in the TCGA ESCA and GSE45670 datasets. (**E**, **F**) The differential expression of 20 m6A-related genes between tumor and normal groups in the TCGA ESCA and GSE45670 datasets. *p < 0.05; **p < 0.01; ***p < 0.001; ns, no significance.

Studies have shown that m6A modification plays a significant role in the regulation of gene expression and signal transduction pathways in various types of tumors. GEPIA online database analysis revealed that DARS2 expression was significantly positively correlated with m6A signatures in ESCA (Figure 7A, p < 0.05). By analyzing the TCGA ESCA and GSE45670 datasets, we investigated the association between DARS2 expression and the expression of 20 m6A-related genes. The correlation analysis results demonstrated a significant positive correlation between DARS2 expression and HNRNPA2B1, METTL3, VIRMA, and YTHDF1 in both TCGA ESCA and GSE45670 datasets (Figure 7B, p < 0.05). Additionally, the expression of the other 16 genes was only significantly positively correlated with DARS2 in the TCGA ESCA dataset. Furthermore, based on the expression levels of DARS2, we divided the TCGA ESCA and GSE45670 datasets into high and low DARS2 expression groups, attempting to analyze the differential expression of m6A-related genes between the high and low DARS2 expression groups in ESCA samples. The results revealed that in the TCGA ESCA dataset, the expression of ALKBH5, FTO, HNRNPA2B1, HNRNPC, IGF2BP1, IGF2BP2, IGF2BP3, METTL14, METTL3, RBM15, RBM15B, RBMX, VIRMA, YTHDF1, YTHDF2, YTHDF3, and ZC3H13 was significantly higher in the high DARS2 expression group compared to the low DARS2 expression group (Figure 7C, p < 0.05). Similarly, in the GSE45670 dataset, the expression levels of METTL3, VIRMA, and YTHDF1 were significantly higher in the high DARS2 expression group compared to the low DARS2 expression group (Figure 7D, p < 0.05), consistent with the analysis results of the TCGA ESCA dataset. Finally, we further analyzed the expression differences of m6A-related genes between tumor samples and normal samples in the TCGA ESCA and GSE45670 datasets to demonstrate the impact of dysregulated expression of m6A-related genes on tumor progression. The results showed that in the TCGA ESCA dataset, compared to the normal group, there was a significant increase in the expression of 16 m6A-related genes in the tumor group, including ALKBH5, FTO, HNRNPA2B1, HNRNPC, IGF2BP1, IGF2BP2, IGF2BP3, METTL3, RBM15, RBMX, VIRMA, WTAP, YTHDC1, YTHDF1, YTHDF2, and YTHDF3 (Figure 7E, p < 0.05). In the GSE45670 dataset, compared to the normal group, there were 11 dysregulated m6A-related genes in the tumor group, including HNRNPA2B1, IGF2BP1, IGF2BP2, IGF2BP3, RBM15, RBM15B, RBMX, YTHDC1, YTHDF1, YTHDF2, and YTHDF3 (Figure 7F, p < 0.05).

### Prediction and construction of the competing endogenous RNA network of DARS2 in ESCA

**Figure 8:**
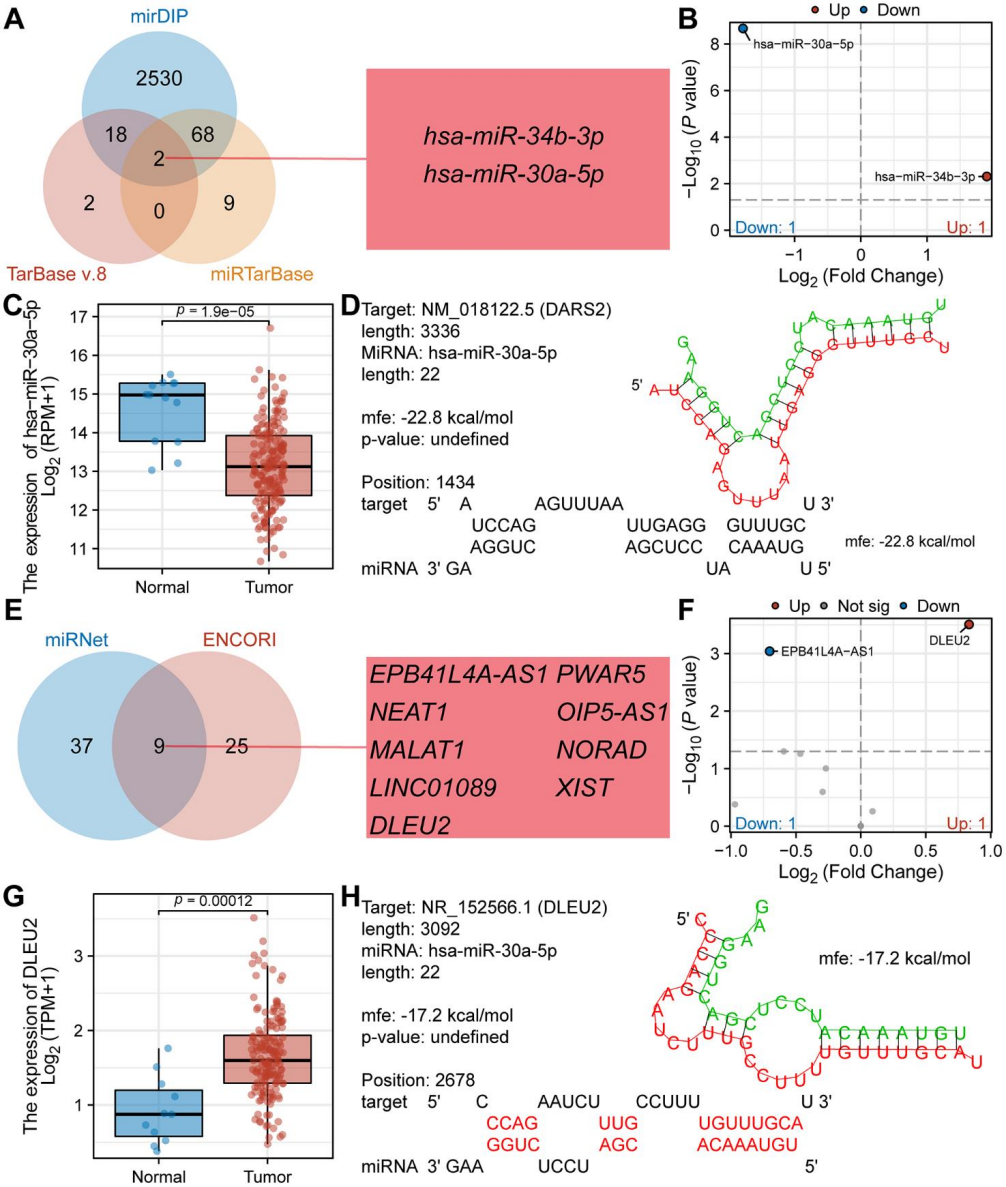
**Prediction and construction of the competing endogenous RNA network of DARS2 in ESCA.** (**A**) The Venn diagram illustrates the co-occurrence of two miRNAs in three databases (TarBase v.8, mirDIP, and miRTarBase). (**B**) The TCGA ESCA dataset showcases the differential expression of the aforementioned two miRNAs. (**C**) The expression of hsa-miR-30a-5p in tumor samples from the TCGA ESCA dataset is significantly lower than that in normal samples. (**D**) Potential binding sites between DARS2 and hsa-miR-30a-5p were predicted using the RNAHybrid online tool. (**E**) The Venn diagram demonstrates the co-occurrence of nine lncRNAs in two databases. (**F**) The TCGA ESCA cohort presents the differential expression of the aforementioned nine lncRNAs. (**G**) The expression of DLEU2 in tumor samples from the TCGA ESCA dataset is significantly higher than that in normal samples. (**H**) Potential binding sites between hsa-miR-30a-5p and DLEU2 were predicted using the RNAHybrid online tool.

Research has shown that abnormal regulation of ceRNA networks may be involved in the occurrence and development of tumors. In this study, we predicted and constructed a ceRNA network associated with DARS2 in ESCA. Using TarBase v.8, mirDIP and miRTarBase databases, we predicted 22, 2618, and 79 miRNAs interacting with DARS2, respectively. The Venn diagram analysis revealed that two miRNAs, hsa-miR-34b-3p and hsa-miR-30a-5p, coexisted in all three databases (Figure 8A). Figure 8B showed the expression differences of hsa-miR-34b-3p (log2FC = 1.90, p = 0.005) and hsa-miR-30a-5p (log2FC = -1.78, p = 2.14E-09) in the TCGA ESCA dataset. According to the ceRNA hypothesis, when mRNA is overexpressed in tumors, corresponding miRNAs should be underexpressed. Therefore, we selected hsa-miR-30a-5p as the targeting miRNA for DARS2. Figure 8C displayed a significant decrease in the expression of hsa-miR-30a-5p in tumor samples compared to normal samples. By utilizing RNAHybrid for bioinformatics analysis, potential binding sites between DARS2 and hsa-miR-30a-5p were identified (Figure 8D). After analyzing the miRNet and ENCORI databases, we predicted 46 and 34 lncRNAs interacting with hsa-miR-30a-5p, respectively. Among them, 9 lncRNAs were found in both databases, namely EPB41L4A-AS1, PWAR5, NEAT1, OIP5-AS1, MALAT1, NORAD, LINC01089, XIST, and DLEU2 (Figure 8E). Differential analysis revealed that only 2 lncRNAs exhibited expression differences in the TCGA ESCA dataset, namely EPB41L4A-AS1 (log2FC = -0.70, p < 0.001) and DLEU2 (log2FC = 0.83, p < 0.001) (Figure 8F). Based on the ceRNA hypothesis, lncRNAs should be highly expressed in tumors when mRNA is overexpressed. Therefore, we selected DLEU2 as the target lncRNA for hsa-miR-30a-5p. Figure 8G demonstrated that the expression level of DLEU2 in tumor samples was significantly higher than that in normal samples. Bioinformatics analysis using RNAHybrid revealed potential binding sites between DLEU2 and hsa-miR-30a-5p (Figure 8H). These data suggest that DLEU2 may act as a competitive ceRNA binding to hsa-miR-30a-5p and promote the expression of DARS2. Our study provides new insights into the role of DARS2 and its ceRNA network in ESCA.

## DISCUSSION

Esophageal cancer is one of the most common cancers, with a high mortality rate worldwide due to its difficulty in early diagnosis and lack of effective treatment [1–3]. In recent years, despite the emergence of minimally invasive esophagectomy, neoadjuvant radiotherapy and chemotherapy, targeted therapy, immunotherapy and other treatment methods, and the progress of these multimodal treatments has shown promising results, they still fail to meet expectations [39], and a considerable number of patients fail to benefit. In the current form, with the growth and aging of the population, the huge burden of new ESCA cases may continue to increase. Therefore, we need to conduct a detailed analysis of the molecular mechanisms of esophageal cancer and identify the therapeutic targets of ESCA as soon as possible, which is of great help in improving the survival rate of esophageal cancer patients.

Mitochondrial oxidative phosphorylation is the main source of energy for normal differentiation cells to rely on to generate cell processes. On the contrary, cancer cells primarily rely on aerobic glycolysis, a phenomenon commonly referred to as the “Warburg effect”. This “Warburg effect” indicates that the characteristics of cancer cells are changes in energy metabolism and increased glucose uptake [40]. Mitochondria are important organs responsible for cellular energy metabolism, and their normal function is crucial for the normal progression of glycolysis pathways [41]. DARS2, as a mitochondrial tRNA synthase, specializes in aminoacylation of aspartyl tRNA [6]. More importantly, its defects are associated with many neurological and mitochondrial diseases, often accompanied by brainstem and spinal cord involvement and elevated lactate levels (LBSL) in white matter encephalopathy [42–44]. In addition, DARS2 is upregulated in bladder cancer [7], lung adenocarcinoma [8], ovarian cancer [10], and hepatocarcinogenesis [11]. Although the overexpression and carcinogenic function of DARS2 in other cancers have been confirmed, the precise involvement and underlying biological mechanism of DARS2 in ESCA remains inadequately comprehended.

In this study, the comprehensive application of bioinformatics analysis enabled the prediction of DARS2 expression in tumors, while the regulatory capacity of DARS2 in ESCA tumor cells was successfully validated via cellular assays. Through analysis of the TCGA database, we found that DARS2 was significantly higher in esophageal cancer samples than in the control group. This finding implies that DARS2 could potentially be a therapeutic target for esophageal cancer, providing a basis for further investigational studies.

By performing co-expression analysis of DARS2 in the TCGA ESCA dataset, we observed a positive correlation between the expression of DARS2 and CENPL, with the highest correlation coefficient. CENPL, a crucial member of the centromere protein family, plays a vital role in cell division and is associated with various human diseases [45, 46]. Previous studies have demonstrated significantly elevated expression of CENPL in liver, breast, and pancreatic cancer samples compared to normal samples, suggesting that high expression of CENPL may serve as a potential prognostic indicator for patients with liver, breast, and pancreatic cancers [47–50]. Additionally, Gui et al. found that downregulation of the CENPL gene could reduce the proliferation and migration abilities of breast cancer cells [51]. Moreover, we also observed a negative correlation between the expression of DARS2 and CBFA2T3, with the highest correlation coefficient. CBFA2T3, an important transcription factor, is involved in the regulation of multiple biological processes. Increased expression of CBFA2T3 has been observed in B Cell Precursor Acute Lymphoblastic Leukemia (BCP-ALL), and the truncation protein of CBFA2T3 significantly decreases BCP-ALL lymphocyte proliferation [52]. Brown et al. [53] discovered that CBFA2T3 serves as a critical regulator of cell fate determination during colon homeostasis, colitis, and cancer by inhibiting the target of the E protein. However, there is currently no report on the relationship between CENPL, CBFA2T3, and ESCA. Subsequently, we performed GO and KEGG enrichment analysis on the co-expression gene set of DARS2 and identified several significant categories enriched in the positively correlated group, including chromosome segregation, chromosome regions, catalytic activity, and nucleocytoplasmic transport. Additionally, GSEA analysis revealed the involvement of differentially expressed genes of DARS2 in various signaling pathways, such as WP_INTEGRATED_CANCER_PATHWAY, REACTOME_REGULATION_OF_TP53_ACTIVITY, REACTOME_MAP2K_AND_MAPK_ACTIVATION, KEGG_CELL_CYCLE, WP_G1_TO_S_CELL_CYCLE_CONTROL, and REACTOME_GLYCOLYSIS. These results imply that DARS2 may play an important biological role in the occurrence and progression of tumors. In this study, we specifically focus on the relationship between DARS2 and tumor cell proliferation, migration, cell cycle, glycolysis, m6A, and ceRNA.

In addition, we also conducted cell experiments *in vitro* to confirm that the mRNA levels, cell activity, EDU, clone count, and wound healing of the two interference groups were significantly reduced after DARS2 was knocked down. The absence of DARS2 inhibited tumor growth and migration. At the same time, it can also be seen that the silenced DARS2 increased the apoptosis rate, indicating that knocking down DARS2 can significantly promote tumor cell apoptosis. Cell cycle analysis revealed that compared to the control group, tumor cells in the experimental group were arrested at the S and G2/M phases, indicating that sustained expression of DARS2 may disrupt the normal cell cycle progression in tumor cells. Although it has been concluded that the expression level of DARS2 may affect cell proliferation, migration, and apoptosis, we still need to further elucidate its mechanism, as more and more people believe that most cancer deaths are caused by metastasis rather than local tumor growth [54]. Cancer cells have many factors that promote metastasis, and our focus is on glycolysis. Proliferating cells prefer aerobic glycolysis, and DARS2 is preferentially expressed in proliferating cells, including cancer cells. We speculate that DARS2 is highly likely to promote aerobic glycolysis of ESCA, which has been confirmed in our subsequent experiments. Firstly, we found that DARS2 was enriched in the glycolytic pathway. During subsequent *in vitro* cell experiments, we observed a significant decrease in the utilization rates of glycolysis and lactate production. Finally, a qRT-PCR experiment was conducted, and the knockdown of DARS2 inhibited the expression of glycolysis-related genes. These results further emphasize the carcinogenic effect of DARS2 and its therapeutic potential in ESCA.

Furthermore, the association of DARS2 with the m6A and ceRNA networks drives us to delve deeper into these two aspects. M6A modification is a common form of RNA methylation modification, which as part of methylation modifications, can affect cancer development by regulating cancer-related biological functions [55–57]. Based on bioinformatics analysis, we propose that the oncogenic effect of DARS2 gene is associated with m6A modification, which may modulate the methylation level of ESCA through its interaction with genes such as METTL3 and YTHDF1, ultimately affecting the progression of ESCA. Interestingly, in our previous research, we have demonstrated that the overexpression of METTL3 and YTHDF1 serve as diagnostic and therapeutic biomarkers in ESCA [58, 59]. In the future, we will further investigate the potential associations between METTL3 and YTHDF1 with DARS2.

Numerous studies have demonstrated the existence of ceRNA networks in many tumor cells, wherein ceRNAs can regulate the expression of other target genes, suggesting a crucial role of the ceRNA mechanism in tumor initiation and progression. Lu et al. [60] identified the upregulation of lncRNA DLEU2 in ESCA tissues, which was significantly associated with poor prognosis. Silencing of lncRNA DLEU2 inhibited the proliferation, migration, and invasion of ESCA cells. Furthermore, the study revealed that lncRNA DLEU2 may serve as a ceRNA sponge for miR-30e-5p, thus influencing E2F7 expression and promoting ESCA progression. Additionally, previous research has revealed that hsa-miR-30a-5p exhibits significantly lower expression levels in lung adenocarcinoma, renal carcinoma, prostate cancer, and hepatocellular carcinoma compared to normal tissues, highlighting its crucial role as a key component of the ceRNA network in cancer development [61–64]. However, to date, no studies have reported on the relationship between DARS2 and ceRNAs in ESCA. In the present study, we initially screened miRNAs interacting with DARS2 using various databases, and performed differential analysis and target prediction to identify hsa-miR-30a-5p as a potential target for DARS2. Subsequently, we predicted a lncRNA (DLEU2) that could potentially target the identified miRNA. Based on the ceRNA theory, we selected and confirmed DLEU2 as a lncRNA that targets hsa-miR-30a-5p, and ultimately constructed a ceRNA network, namely DLEU2/has-miR-30a-5p/DARS2. This study provides the first prediction and proposal of a ceRNA network targeting DLEU2 in ESCA, thereby offering potential therapeutic targets for molecular treatment of ESCA.

## CONCLUSIONS

In conclusion, our study establishes a novel connection between DARS2 and ESCA, but further experimental validation and larger sample sizes are required to comprehensively confirm our hypothesis. Specifically, it is crucial to investigate the impact of DARS2 on cell proliferation and migration in ESCA and examine its relationship with the glycolysis pathway. Our findings reveal the upregulation of DARS2 in ESCA and its association with clinical characteristics, the glycolysis pathway, m6A modifications, and ceRNA networks. This study enhances our understanding of the molecular mechanisms underlying ESCA and sets the stage for future investigations into targeted therapies involving DARS2 manipulation.

## Supplementary Material

Supplementary Table 1
