# Supplementary material for: Functional enrichment analysis reveals the involvement of DARS2 in multiple biological pathways and its potential as a therapeutic target in esophageal carcinoma
Source: Aging (Albany NY). 2024 Feb 20;16(4):3934–54. doi: 10.18632/aging.205569 (PMC10929822; doi:10.18632/aging.205569)
Supplement: Supplementary Table 1 [file aging-16-205569-s001.pdf]

## SUPPLEMENTARY TABLE

**Supplementary Table 1. The sequences of primers used in this study.**

| Gene | Forward primer sequences | Reverse primer sequences |
|---|---|---|
| β-actin | TGGCACCCAGCACAATGAA | CTAAGTCATAGTCCGCCTAGAAGCA |
| DARS2 | CATGGAGGAATTGCCTTAGGGTTA | ATGTCCCCGGAAGGACTTTG |
| SLC2A1 | CACTGTCGTGTCGCTGTTTG | CTAGCGCGATGGTCATGAGT |
| HK2 | GGCAAGCAGAGGTTCGAGA | AAGTGTTGCAGGATGGCTCG |
| GPI | AAGGGTCTGCATCACAAGATCC | AGAGTTGGTTGGGCGATTTCC |
| PFKL | GAGGTTTACCGCAAGGGACG | ACTGATGCGGTATTGTGCCA |
| ALDOA | ACATCGCTCACCGCATCGT | GGTAGTCTCGCCATTTGTCCC |
| GAPDH | GAGAAGGCTGGGGCTCATTT | GCAGGAGGCATTGCTGATGA |
| PGK1 | TGGAGCTCCTGGAAGGTAAAG | GTTCCTGGCACTGCATCTCT |
| PGAM1 | TCAATGAGCGGCACTATGGG | TGCTGTAGAAAGGATGGTCGG |
| ENO1 | TGTGCACTGGGCAGATCAAG | GCGCTAACTAGCAGGGACC |
| PKM2 | GCTCCGGATCTCTTCGTCTT | GATGGTCTCCGCATGGTACT |
| LDHA | ACGACCGCCCGACGTG | GCAAGTTCATCTGCCAAGTCCTTC |
